# Faster insulin action is associated with improved glycaemic outcomes during closed‐loop insulin delivery and sensor‐augmented pump therapy in adults with type 1 diabetes

**DOI:** 10.1111/dom.12956

**Published:** 2017-06-23

**Authors:** Yue Ruan, Hood Thabit, Lalantha Leelarathna, Sara Hartnell, Malgorzata E. Wilinska, Martin Tauschmann, Sibylle Dellweg, Carsten Benesch, Julia K. Mader, Manuel Holzer, Harald Kojzar, Mark L. Evans, Thomas R. Pieber, Sabine Arnolds, Roman Hovorka, on behalf of AP@home consortium

**Affiliations:** ^1^ Wellcome Trust‐MRC Institute of Metabolic Science University of Cambridge Cambridge UK; ^2^ Department of Paediatrics University of Cambridge Cambridge UK; ^3^ Department of Diabetes & Endocrinology Cambridge University Hospitals NHS Foundation Trust Cambridge UK; ^4^ Profil Neuss Germany; ^5^ Division of Endocrinology and Diabetology, Department of Internal Medicine Medical University of Graz Graz Austria

**Keywords:** CSII, glycaemic control, insulin delivery, insulin pump therapy, pharmacodynamics, type 1 diabetes

## Abstract

We aimed to evaluate the relationship between insulin pharmacodynamics and glycaemic outcomes during closed‐loop insulin delivery and sensor‐augmented pump therapy. We retrospectively analysed data from a multicentre randomized control trial involving 32 adults with type 1 diabetes receiving day‐and‐night closed‐loop insulin delivery and sensor‐augmented pump therapy over 12 weeks. We estimated time‐to‐peak insulin action (t_max,_ _IA_) and insulin sensitivity (S _I_) during both interventions, and correlated these with demographic factors and glycaemic outcomes. During both interventions, t_max,_ _IA_ was positively correlated with pre‐ and post‐intervention HbA1c (r = 0.50‐0.52, P < .01) and mean glucose (r = 0.45‐0.62, P < .05), and inversely correlated with time sensor glucose, which was in target range 3.9 to 10 mmol/L (r = −0.64 to −0.47, P < .05). Increased body mass index was associated with higher t_max,_ _I_ and lower S _I_ (both P < .05). During closed‐loop insulin delivery, t_max,_ _IA_ was positively correlated with glucose variability (P < .05). Faster insulin action is associated with improved glycaemic control during closed‐loop insulin delivery and sensor‐augmented pump therapy.

## 1 INTRODUCTION

Rapid‐acting insulin analogues are widely used in insulin pump‐treated type 1 diabetes and closed‐loop insulin delivery systems.^1^ However, little is known about the association between its pharmacodynamics, and demographic factors and glycaemic outcomes during closed‐loop insulin delivery and sensor‐augmented pump therapy.

## 2 METHODS

We retrospectively analysed data obtained from a multicentre (UK, Germany and Austria), randomized crossover study involving 32 participants with type 1 diabetes and conducted in free‐living home settings.^2^ Participants were randomly assigned to receive 12 weeks of automated closed‐loop insulin delivery first and sensor‐augmented pump therapy (open‐loop) second, or vice versa applying rapid‐acting insulin analogue, aspart or lispro, to follow their pre‐study insulin use. Day‐and‐night closed‐loop insulin delivery was applied using a hybrid approach, during which participants administered prandial insulin using standard pump bolus wizard. The participants underwent 4 to 6 weeks of run‐in period using the study insulin pump and real‐time continuous glucose monitoring device prior to randomization to fully optimize insulin delivery.

Using a validated modelling approach analysing continuous glucose monitoring, insulin delivery and meal content data (outlined in File S1),^3^ we estimated time‐to‐peak insulin action (*t*_max,IA_; representing time to maximum insulin action) and insulin sensitivity (*S*_I_; representing glucose‐lowering potency of insulin) during closed‐loop and open‐loop interventions. The approach utilized compartment modelling of insulin absorption and action, meal absorption dynamics and glucose dynamics. Parameters were estimated using Bayesian estimator and checked for normality. The validity of the model was evidenced by the physiological plausibility of model parameters, good model fit and the ability to reproduce independent clinical data.^3^ Pearson correlation coefficient was used to relate these model‐derived parameters, demographic factors and glycaemic outcomes, which included age, body mass index (BMI), pre‐ and post‐intervention HbA1c, mean glucose, time with sensor glucose in the target range between 3.9 and 10 mmol/L, and glucose variability expressed as the coefficient of variation (CV). *P*‐values less than .05 were considered statistically significant. Statistical analyses were performed using SPSS (IBM Software, Hampshire, UK, version 21). Data are reported as mean (SD), unless stated otherwise.

## 3 RESULTS

Data from 32 adults with type 1 diabetes [male 17, age 39.9 (9.5) years, BMI 25.4 (4.4) kg/m^2^, duration of diabetes 21.2 (9.3) years, duration of pump use 7.9 (6.0) years] were analysed. We estimated time‐to‐peak insulin action and insulin sensitivity in 32 closed‐loop participants and 28 open‐loop participants; four open‐loop participants’ datasets were excluded from the final analysis due to insufficient data (less than 50 days of continuous glucose‐monitoring data) deemed appropriate for accurate subject‐level parameter estimates. Time‐to‐peak insulin action and insulin sensitivity were 79 (12) minutes and 4.7 (1.2) 10^−3^ mM/min per mU/L during the closed‐loop intervention, and 72 (14) minutes and 4.2 (1.1) 10^−3^ mM/min per mU/L during the open‐loop intervention. No statistically significant differences were observed between parameters estimated during open‐loop and closed‐loop interventions (Table S1, File S1), supporting the validity of the estimates.

**Figure 1:**
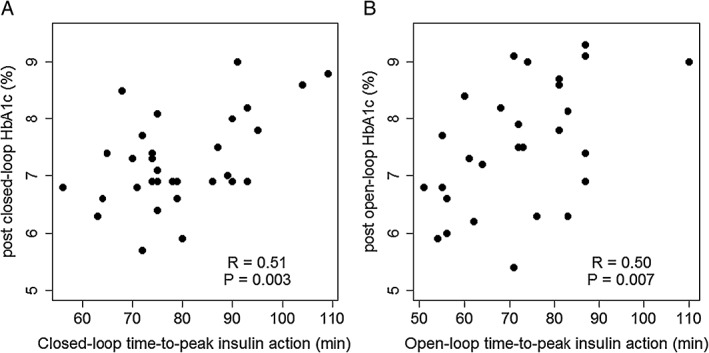
Time‐to‐peak insulin action (t_max,_ _IA_) vs post‐intervention HbA1c during closed‐loop insulin delivery (A) and sensor‐augmented pump therapy (B)

Table 1 reports the Pearson correlation coefficients between time‐to‐peak insulin action and insulin sensitivity, and demographic factors and glycaemic outcomes. During both interventions, time‐to‐peak insulin action was positively correlated with pre‐ and post‐intervention HbA1c (*P* < .01) and mean glucose levels (*P* < .05‐.01), whilst being inversely correlated with time sensor glucose, which was in target range of 3.9 to 10 mmol/L (*P* < .05‐.01). A higher BMI was associated with higher time‐to‐peak insulin action (*P* < .05‐.01) and lower insulin sensitivity (*P* < .05). A positive correlation was observed between time‐to‐peak insulin action and glucose variability during closed‐loop (*P* < .05) but not during open‐loop intervention. No other relationship was observed. Figure 1 shows scatter plots of time‐to‐peak insulin action vs post‐intervention HbA1c for the 2 interventions.

**Table 1:** Pearson correlation between parameters of glucose–insulin regulation, and demographic factors and glycaemic outcomes

	Age (years)		BMI (kg/m^2^)		HbA1c pre‐intervention (%)		HbA1c post‐intervention (%)		Mean glucose (mmol/L)		Glucose time in target (3.9‐10 mmol/L) (%)		Glucose variability, CV (%)	
	CL	OL	CL	OL	CL	OL	CL	OL	CL	OL	CL	OL	CL	OL
Time‐to‐peak insulin action (min)	0.02	0.16	**0.46** **	**0.41** *	**0.50** **	**0.52** **	**0.51** **	**0.50** **	**0.62** **	**0.45** *	−**0.64** **	−**0.47** *	**0.43** *	0.19
Insulin sensitivity (mM/min per mU/L)	0.22	−0.06	−**0.36** *	−**0.41** *	−0.04	−0.12	−0.16	−0.07	−0.10	−0.12	0.13	0.10	−0.03	0.21

Abbreviations: BMI, body mass index; CL, closed‐loop intervention; OL, open‐loop intervention. Significant correlations are shown in boldface type.

* P < .05;

** P < .01.

## 4 DISCUSSION

We estimated time‐to‐peak insulin action and insulin sensitivity in adults with type 1 diabetes during 12‐week closed‐loop insulin delivery and conventional insulin pump therapy, and demonstrated associations with clinical factors of interest.

Time‐to‐peak insulin action reflects the timespan for the subcutaneously delivered rapid‐acting insulin to reach the peak glucose‐lowering effect. The population estimates 79 minutes during the closed‐loop intervention and 72 minutes during sensor‐augmented insulin therapy, which compares well with 90 to 100 minutes^4^ measured during glucose clamp studies when higher insulin doses were administered resulting in endogenous glucose production to be maximally suppressed and the measurements reflecting primarily slower insulin action through augmentation of glucose disposal in the muscle. During both interventions, significant correlations were observed between time‐to‐peak insulin action and glycaemic outcomes including pre‐ and post‐intervention HbA1c, mean glucose and percentage of time spent with glucose in the target range. These correlations suggest that faster insulin absorption may be associated with improved glycaemic control, and that acceleration may provide further benefit. The observation is in agreement with previous findings that showed that treatment with rapid‐acting insulin analogues in type 1 diabetes resulted in improved glucose control compared with regular human insulin,^5^, ^6^, ^7^ even under the condition that regular human insulin meal time bolus was titrated 30 minutes ahead of meals whilst rapid‐acting insulin bolus was titrated at meal time. The sole difference between rapid‐acting insulin and regular human insulin is the faster insulin absorption and thus action. A recent trial evaluated the efficacy of faster‐acting insulin aspart and demonstrated a greater reduction in HbA1c (−0.15%) for meal time faster aspart compared with insulin aspart after 26 weeks treatment.^8^ The time‐to‐maximum plasma insulin concentration of faster‐acting insulin aspart is 26 minutes left‐shifted compared with that of insulin aspart during insulin pump therapy.^9^ The highly significant correlation between time‐to‐peak insulin action and mean glucose levels accounts for about 40% of the between‐subject variability in glucose levels.

A positive correlation between time‐to‐peak insulin action and glucose variability was observed during closed‐loop but not open‐loop intervention. The clinical significance of the correlation might have been “diluted” during open‐loop as basal insulin delivery was less variable. The higher variability of basal insulin delivery during closed‐loop accentuated the importance of faster insulin absorption and action.

Faster insulin action was associated with a lower BMI. A similar trend was previously reported between subcutaneous absorption of insulin aspart and BMI.^10^ Increasing subcutaneous adiposity is expected to result in reduction of subcutaneous blood flow and the rate of absorption of rapid‐acting insulin and the peak time of insulin action.

In our previous study, we reported the variability of individual insulin requirements during closed‐loop intervention.^11^ When time‐to‐peak insulin action was correlated with variability of insulin requirements, slower insulin action was found to be associated with more variable overnight insulin requirements (*r* = .55, *P* = .001). This suggests that slower insulin action may provide a further challenge to optimize overnight insulin rate during conventional pump therapy.

Insulin sensitivity was estimated at 0.0047 and 0.0042 mM/min per mU/L during the closed‐loop and open‐loop interventions, respectively. These estimates are in concordance with published data reporting 0.0005/min per mU/L at a glucose concentration of 8 to 10 mmol/L.^12^ However, we have not compared our estimates of insulin sensitivity with those obtained with the gold standard euglycaemic hyperinsulinaemic clamp test. We observed a negative correlation between insulin sensitivity and BMI in agreement with previously reported data in adults with type 1 diabetes.^13^

The main novelty of the present study is the finding of the positive correlation between time‐to‐peak insulin action and HbA1c level, which highlights the need for new insulin formulations or other novel delivery methods that could result in faster insulin absorption and action. The development of faster‐acting insulin aspart,^9^ inhaled insulin^14^ and infusion site warming devices^15^ may contribute towards this goal. These new formulations and delivery methods may benefit both conventional insulin pump therapy and the closed‐loop insulin delivery system.

In conclusion, faster insulin action was associated with better glycaemic control during closed‐loop insulin delivery and sensor‐augmented pump therapy, justifying further research to be directed towards accelerating insulin absorption and action.

## Supporting information


**Table S1.** Time‐to‐peak insulin action and insulin sensitivity during closed‐loop insulin delivery and sensor augmented pump therapy.
