# Supplementary material for: Faster insulin action is associated with improved glycaemic outcomes during closed‐loop insulin delivery and sensor‐augmented pump therapy in adults with type 1 diabetes
Source: Diabetes Obes Metab. 2017 Jun 23;19(10):1485–9. doi: 10.1111/dom.12956 (PMC5638091; doi:10.1111/dom.12956)
Supplement: Supplementary file 1 — Table S1. Time‐to‐peak insulin action and insulin sensitivity during closed‐loop insulin delivery and sensor augmented pump therapy. [file DOM-19-1485-s001.docx]

**Supplemental Material**

**Table S1. Time-to-peak insulin action and insulin sensitivity during closed-loop insulin delivery and sensor augmented pump therapy.**

|  | Closed-loop* | Open-loop* | P-value** |
| --- | --- | --- | --- |
| Time-to-peak insulin action (min) | 79(12) | 72(14) | 0.12 |
| Insulin sensitivity  (x10^-3^ mmol/l/min per mU/l) | 4.7(1.2) | 4.2(1.1) | 0.15 |

* Values are mean (sd)

** Paired t-test

**Estimation of time-to-peak insulin action and insulin sensitivity**

Time-to-peak insulin action (t_max,IA_) and insulin sensitivity (S_I_) were estimated using compartment modelling within a Bayesian hierarchical framework (1).

***Model description***

The model comprises seven parameters including t_max,IA_ and S_I_. It consists of three sub-models describing insulin absorption and action, meal absorption dynamics and glucose dynamics. The model uses subcutaneous insulin delivery and carbohydrate intake as model input and continuous glucose monitoring as model output. The model is described as follow.

### *Insulin Absorption and Action*

The insulin absorption and action sub-model is described by a set of equations:

$$\frac{dx_{1}(t)}{dt}=-\frac{1}{t_{\max,IA}}x_{1}(t)+\frac{u_{I}(t)}{60}$$

$$\frac{dx_{2}(t)}{dt}=\frac{1}{t_{\max,IA}}(x_{1}(t)-x_{2}(t))$$

$$X(t)=\frac{1000\cdot x_{2}(t)}{t_{\max,IA}MCR_{I}W}$$

where *x_1_* (*t*) and *x_2_* (*t*) represent the amount of effective insulin in the first and second insulin absorption compartment, respectively (U); *u_I_* (*t*) represents exogenous delivery rate of insulin aspart (U/h) at time *t* (immediate insulin bolus is modelled as a short burst insulin infusion); *t_max,IA_* is the time-to-maximum of effective insulin concentration (min); *X* (*t*) is the concentration of effective insulin (mU/l); $W$ is subject’s body weight (kg); and *MCR_I_* is the metabolic clearance rate of effective insulin fixed at 0.017 (l/kg/min).

### *Meal Absorption Dynamics*

The meal absorption is represented by two compartments and described by the following equations:

$$\frac{da_{1}(t)}{dt}=-\frac{1}{t_{\max,G}}a_{1}(t)+\delta_{t_{j}}(t)u_{G}(t_{j})$$

$$\frac{da_{2}(t)}{dt}=\frac{1}{t_{\max,G}}(a_{1}(t)-a_{2}(t))$$

$$U_{M}(t)=\frac{5.556\cdot A_{G}a_{2}(t)}{t_{\max,G}V_{G}W}$$

where *a_1_*(*t*) and *a_2_*(*t*) represent carbohydrate amount in the first and second meal absorption compartment, respectively (g); *u_G_*(*t_j_*) represents the carbohydrate amount eaten at time *t_j_* (g); *t_max,G_* is the time-of-maximum appearance rate of glucose (min); *A_G_* is the fractional bioavailability (unitless); *V_G_* is the plasma glucose pool size fixed at 0.16 (l/kg); *U_M_* (*t*) is the gut carbohydrate absorption rate with unit converted to glucose concentration rate of change (mmol/l/min).

### *Glucose Dynamics*

The kinetics of the continuously monitored glucose concentration is represented by a single compartment:

$$\frac{dG(t)}{dt}=-S_{I}(X(t)-X_{b})+U_{M}(t)-K(G(t)-G_{b})$$

where *G* (*t*) is the blood glucose concentration (mmol/l); *S_I_* is the insulin sensitivity (mmol/l/min per mU/l); *X_b_* represents the basal effective insulin concentration at which glucose level would be maintained constant (mU/l); *G_b_* is the basal glucose level (mmol/l) and *K* the glucose self-regulation fractional rate (/min) which has an effect of self-regulating the glucose level towards *G_b_*.

***Parameter estimation***

To estimate individual model parameters, a multi-day dataset with up to 84 days per subject was used by a Bayesian hierarchical approach which incorporated vague prior information on the parameters at the subject-level. For each subject, estimates of the day-level parameters t_max,IA_, S_I_ and others (one set of parameters per day; during the day parameters were assumed time invariant) were made in parallel with the subject-level mean. Subject-level estimates of t_max,IA_ and S_I_ were used in the present analysis.

**Reference**

1. Ruan Y, Wilinska ME, Thabit H, Hovorka R: Modelling Day-to-Day Variability of Glucose-Insulin Regulation over 12-Week Home Use of Closed-Loop Insulin Delivery. IEEE Trans Biomed Eng 2016; DOI: 10.1109/TBME.2016.2590498
